# Parallel Evaluation of Polyethylene Glycol Conformal Coating and Alginate Microencapsulation as Immunoisolation Strategies for Pancreatic Islet Transplantation

**DOI:** 10.3389/fbioe.2022.886483

**Published:** 2022-05-16

**Authors:** Teresa De Toni, Aaron A. Stock, Floriane Devaux, Grisell C. Gonzalez, Kailyn Nunez, Jessica C. Rubanich, Susan A. Safley, Collin J. Weber, Noel M. Ziebarth, Peter Buchwald, Alice A. Tomei

**Affiliations:** ^1^ Diabetes Research Institute, University of Miami Miller School of Medicine, Miami, FL, United States; ^2^ Department of Biomedical Engineering, University of Miami, Miami, FL, United States; ^3^ Department of Surgery, Emory University, Atlanta, GA, United States; ^4^ Department of Molecular and Cellular Pharmacology, University of Miami, Miller School of Medicine, Miami, FL, United States; ^5^ Department of Surgery, University of Miami Miller School of Medicine, Miami, FL, United States; ^6^ Department of Microbiology and Immunology, University of Miami Miller School of Medicine, Miami, FL, United States

**Keywords:** alginate, polyethylene glycol, capsules, type 1 diabetes, encapsulation, transplantation

## Abstract

Pancreatic islet transplantation improves metabolic control and prevents complications in patients with brittle type 1 diabetes (T1D). However, chronic immunosuppression is required to prevent allograft rejection and recurrence of autoimmunity. Islet encapsulation may eliminate the need for immunosuppression. Here, we analyzed in parallel two microencapsulation platforms that provided long-term diabetes reversal in preclinical T1D models, alginate single and double capsules versus polyethylene glycol conformal coating, to identify benefits and weaknesses that could inform the design of future clinical trials with microencapsulated islets. We performed *in vitro* and *in vivo* functionality assays with human islets and analyzed the explanted grafts by immunofluorescence. We quantified the size of islets and capsules, measured capsule permeability, and used these data for *in silico* simulations of islet functionality in COMSOL Multiphysics. We demonstrated that insulin response to glucose stimulation is dependent on capsule size, and the presence of permselective materials augments delays in insulin secretion. Non-coated and conformally coated islets could be transplanted into the fat pad of diabetic mice, resulting in comparable functionality and metabolic control. Mac-2^+^ cells were found in conformally coated grafts, indicating possible host reactivity. Due to their larger volume, alginate capsules were transplanted in the peritoneal cavity. Despite achieving diabetes reversal, changes in islet composition were found in retrieved capsules, and recipient mice experienced hypoglycemia indicative of hyperinsulinemia induced by glucose retention in large capsules as the *in silico* model predicted. We concluded that minimal capsule size is critical for physiological insulin secretion, and anti-inflammatory modulation may be beneficial for small conformal capsules.

## Introduction

In autoimmune diseases, such as type 1 diabetes (T1D), Hashimoto’s thyroiditis, lupus, and inflammatory bowel disease, the immune response is directed against self-antigens, and it results in inflammation and destruction of healthy self-tissues (Wang et al., 2015). Specifically, in T1D or *insulin-dependent diabetes*, the immune system attacks and destroys the insulin-producing β cells found in the pancreatic islets of Langerhans (Copenhaver and Hoffman, 2017; Katsarou et al., 2017). This leads to severe and chronic hyperglycemia and requires life-long glycemic monitoring and administration of exogenous insulin multiple times a day. Despite improvements in the treatment of T1D, the disease is associated with an increased risk of long-term complications such as blindness, renal failure, heart attacks, stroke, limb amputation, and even death (Katsarou et al., 2017).

Pancreatic islet transplantation is an emerging and promising cell therapy for T1D. It is a minimally invasive procedure that ameliorates metabolic control and quality of life, while reducing hypoglycemia unawareness and long-term complications. Islets of Langerhans are infused through the portal vein of the liver, allowing for the reestablishment of physiological insulin secretion (Shapiro, 2012; Shapiro et al., 2017). However, the procedure requires the administration of systemic lifelong immunosuppression to prevent allograft rejection and recurrence of autoimmunity against the transplanted islets that would otherwise lead to loss of islet graft functionality. Unfortunately, the prolonged use of immunosuppressive treatments can cause undesired side effects, including organ toxicity and increased susceptibility to infections and malignancies. For this reason, islet transplantation is currently limited to patients who suffer from severe and unstable T1D, have multiple episodes of hypoglycemia unawareness/glycemic lability, and are not capable of stabilizing the disease even with intensive insulin treatment including pumps and/or glucose-monitoring therapies (Shapiro, 2012; Mohsen et al., 2015; Hering et al., 2016; Shapiro et al., 2017; Markmann et al., 2021).

The encapsulation of islets in natural or synthetic biomaterials emerged during the 1980s as a strategy to eliminate the need for systemic and chronic immunosuppressive therapies. Capsules are hydrogels that form an immune-protective shield around the islets and prevent their contact with host immune cells (Foster and García, 2017; Basta et al., 2021). The ideal hydrogel for islet encapsulation is permeable to essential nutrients such as oxygen and glucose as well as the released insulin, while it prevents contact between the transplanted cells and the host immune system. Eliminating the need for lifelong systemic immunosuppressive therapy would increase the safety and the efficiency of islet transplantation, extending its applicability to a larger number of patients with T1D.

So far, several encapsulation technologies have reversed diabetes in preclinical models, but none has yet demonstrated long-term efficacy in large animal models and humans (Dimitrioglou et al., 2019). Parallel evaluation of different encapsulation platforms in the same experimental setting, which could provide critical information to identify strengths and weaknesses to be addressed before clinical testing, has not been performed. Here, we aimed at examining in parallel selected microencapsulation platforms that were previously reported to permit long-term diabetes reversal in preclinical T1D models: single and double capsules (SCs, traditional microencapsulation method, and DCs, improved method for biocompatibility, respectively) made of alginate (Safley et al., 2013; Safley et al., 2018; Safley et al., 2020) (medium viscosity high guluronic, MVG, or low viscosity high mannuronic acid, LVM) and conformally coated capsules (CCs, improved method for capsule size minimization) made of polyethylene glycol (PEG) (Tomei et al., 2014; Manzoli et al., 2018). We determined how the different capsule characteristics affect the functionality of primary human islets (HIs) *in vitro* (i.e., glucose-stimulated insulin secretion, GSIS) and *in vivo* (blood glucose monitoring and glucose tolerance test, GTT) in their conventional transplant site (SCs, DCs: peritoneal cavity; CCs: fat pad) compared with non-coated (NC) HIs. We also evaluated the grafts histologically after retrieval to determine and compare effects on host responses and islet composition. To connect the observed *in vitro* and *in vivo* functionality results to differences in glucose and insulin transport kinetics between platforms, we measured capsule permeability and quantified the size of islets and capsules, and we used these data to perform *in silico* GSIS simulations through COMSOL Multiphysics. Our results can inform future design modifications to these different microencapsulation platforms to improve their functionality and likelihood of success in clinical trials.

## Materials and Methods

### Cell-Free Capsule Formation

Cell-free alginate SCs were obtained by extruding 2% (w/v) sterile-filtered ultrapure LVM sodium alginate (lot # BP-1606-16, Novamatrix) or 1.2% (w/v) sterile-filtered ultrapure MVG sodium alginate (lot # BP-1103-01, Novamatrix) through a 0.5-mm nozzle into a 50 mM SrCl_2_ gelling solution supplemented with 200 mM mannitol (Sigma) and 25 mM HEPES (Gibco), using an electrostatic droplet generator (Nisco). The alginate extrusion flow rate applied for SC formation was 100 μL/min, and the voltage applied was 7.5 kV. For DCs, SCs were first coated with 50 ml 0.05% (w/v) PLL (poly-L-lysine) (Sigma), cultured overnight, then resuspended in 1.26% LVM or 0.95% MVG alginate, and extruded through a 1.1-mm nozzle, into the gelling solution, at a flow rate of 200 μL/min with 7.5 kV voltage.

Cell-free PEG capsules were obtained manually by suspension of 5% w/v 10 kDa 8arm-PEG-maleimide (75% functionalized with maleimide groups) (Jenkem Technology, Plano, TX) with HS-PEG-SH (2 kDa, PEG-SH, Jenkem) or dithiothreitol (DTT, Sigma) (ratio 1:9) in polypropylene glycol (4k Mn, PPG, Sigma) + 10% Span80 (Sigma) + 0.02% triethanolamine (TEA, Sigma) to obtain 1-mm-diameter capsules. The capsules were gelled for ∼12 min. After gelation, the capsules were separated from PPG through centrifugation, one wash with 5% w/v BSA (Proliant Biologicals), and three washes with HBSS (Sigma).

### Islet Encapsulation

Human islets (HIs) were procured from the Integrated Islet Distribution Program (IIDP) at City of Hope, Prodo Labs, or the cGMP (current Good Manufacturing Practice) Human Islet Cell Processing Facility at the Diabetes Research Institute (DRI), University of Miami (UM), Miami, FL, United States. For the latter, HIs were isolated using a modification of the automated method according to the protocol standardized as part of the Clinical Islet Transplant (CIT) consortium and under the exemption issued by the UM Institutional Review Board (IRB) (Ricordi et al., 1988; Hering et al., 2016). The number of islets was quantified as islet equivalents (IEQ) (Buchwald et al., 2009).

**FIGURE 1:**
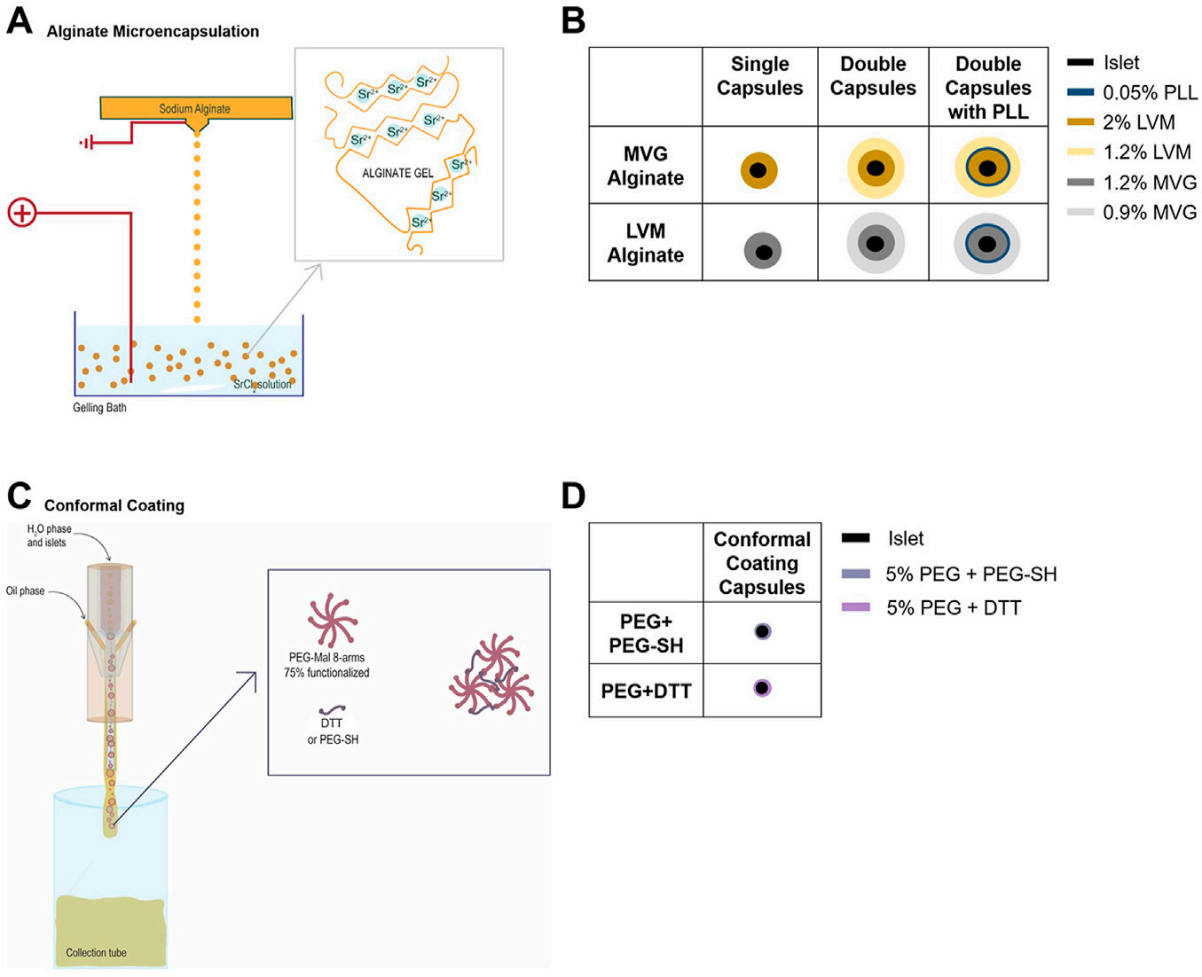
Encapsulation of human islets (HIs) in alginate microcapsules or through polyethylene glycol (PEG) conformal coating (CC). **(A–D)** Electrostatic microdroplet generator **(A)** and schematic of MVG and LVM alginate single (SC) and double capsules (DC) with and without PLL **(B)**. CC device and capsule formation through Michael-type addition reaction **(C)** and schematic of PEG conformal capsules **(D)**.

For alginate microencapsulation (Figures 1A,B), HIs were suspended in 2% (w/v) sterile-filtered ultra-pure LVM or in 1.2% (w/v) sterile-filtered ultra-pure MVG and extruded through a 0.5-mm or 0.7-mm nozzle (depending on the size of the islets) into a SrCl_2_ crosslinking solution, using an electrostatic droplet generator, as for cell-free SCs. After crosslinking, the capsules were divided into three samples for SC analysis 1), and DC formation, with 2) or without 3) PLL. For DCs with 0.05% (w/v) PLL, SCs were cultured for at least 4 h prior to PLL coating and then cultured overnight. The following day, SCs without PLL and SCs with PLL were resuspended in 1.26% (w/v) sterile filtered ultra-pure LVM or 0.95% (w/v) ultra-pure MVG, and extruded through a 1.1-mm nozzle into the SrCl_2_ crosslinking solution. After crosslinking, the capsules were cultured for 24 h prior to testing and counted.

For PEG CC encapsulation (Figures 1C,D), 6.05% (w/v) PEG-MAL was partially crosslinked with 36.2% (w/v) PEG-SH. HIs were resuspended in this viscous solution and extruded through a proprietary CC microfluidic device (Biorep) using a PPG + 10% Span80 external oil solution and 25 mg/ml DTT in HBSS^--^/PPS gelling emulsion that was flowed coaxially. The capsules were cultured for 48 h prior to testing and counted.

### Quantification of Islet and Capsule Size

Phase contrast images were taken with a Leica optical microscope, using a ×10 objective before and after encapsulation. Quantitative analyses were performed using ImageJ (NIH, Bethesda, MD) (Schneider et al., 2012) to evaluate islet diameter (Feret diameter in µm), capsule thickness (distance between the external perimeter of the islet and the external layer of capsules), and capsule diameter (Feret diameter in µm) of ≥3 batches of HI.

### Characterization of Islet Functionality *In Vitro*

HIs encapsulated in CCs, SCs, and DCs with and without PLL were used to evaluate islet functionality. NC HIs were used as controls. Parallel dynamic perifusion GSIS assays were used to evaluate insulin secretion by NC and encapsulated HIs using a PERI4-02 machine (Biorep Technologies, Miami, FL, United States), as previously described (Buchwald et al., 2018). Briefly, for each experiment, 100 IEQ NC or encapsulated HIs (all from the same isolation batch) were handpicked and loaded in Perspex microcolumns between two layers of acrylamide-based microbead slurry (Bio-Gel P-4, Bio-Rad Laboratories, Hercules, CA) by the same experienced operator. Perifusing buffer (114 mM NaCl, 4.72 mM KCl, 2.56 mM CaCl_2_·2H_2_O, 1.2 mM MgCl_2_·7H_2_O, 1.2 mM KH_2_PO_4_, 25 mM HEPES, and 0.2% w/v bovine serum albumin) at 37°C, with glucose (low = 2.2 mM; high = 16.6 mM) or KCl (25 mM) in 26 mM NaCl was circulated through the columns at a rate of 100 μL/min. After 45–60 min of washing with the low glucose solution for stabilization, HIs were stimulated with sequential solutions of low glucose (2.2 mM; 8 min), high glucose (16.6 mM; 20 min), low glucose (2.2 mM; 15 min), depolarizing KCl (30 mM; 10 min), and low glucose (2.2 mM; 10 min). Serial samples (100 μL) were collected every minute from the outflow tubing of the columns in an automatic fraction collector designed for a multi-well plate format. The collected islets and the perifusion solutions were stored at 37°C in a temperature-controlled chamber. The perifusate in the collecting plate was stored at −80°C to preserve the integrity of the analytes. Insulin concentrations were determined with commercially available ELISA kits (Mercodia Inc., Winston Salem, NC). Delays in the insulin response were quantified by comparing the highest peak of each condition after high glucose and KCl stimulation and by calculating the 50% shutdowns of insulin secretion during the transition from high to low. The areas under the curve (AUC) were evaluated to compare the amounts of insulin released using the different encapsulations.

### Characterization of Islet Functionality *In Vivo*

All animal studies were performed under a protocol approved by the University of Miami Institutional Animal Care and Use Committee (IACUC protocol 19-004). Mice were purchased from the Jackson Laboratory (Bar Harbor, ME).

NOD-scid mice were rendered chemically diabetic through five daily intravenous injections of streptozotocin (40 mg/kg). Diabetic animals (>3 blood glucose readings >350 mg/dl) received 2000 IEQ of NC HIs, SC HIs, CC HIs, or 1000 IEQ of DC HIs (with or without PLL). NC and CC islets were resuspended in ∼10 µL of HI media and transplanted in the fat pad (FP) of the mice. The FP was closed and sealed with a biological scaffold formed using autologous plasma from NOD-scid mice and human recombinant thrombin (Recothrom, Zymogenetics, Seattle, WA). SCs and DCs were transplanted into the intraperitoneal cavity (IP) of the mice. Non-fasting blood glucose was monitored to determine diabetes reversal (3 consecutive blood glucose reading <250 mg/dl) for 31, 40, and over 100 days. Hypoglycemic condition was determined as blood glucose levels <50 mg/dl (Park et al., 2012). After 30 days, the mice were subjected to fasting and an intraperitoneal glucose tolerance test (IPGTT). Blood samples were collected at 0, 5, 20, and 30 min after injection of 20% w/v glucose intraperitoneally. The animals were subsequently euthanized and the grafts retrieved.

### Histological Evaluation

Formalin-fixed explanted grafts were embedded in paraffin and sectioned at 5–10 µm thickness. Hematoxylin and eosin (H&E) stained sections were imaged using a Leica optical microscope. For immunofluorescence, cells were stained with DAPI (1:10,000, Invitrogen, D1306) and antibodies specific for insulin (1:100, Dako, cat A0564), glucagon (1:250, Biogenex, cat PU039-UP), Mac-2 (1:100, Cederlane, cat CL8942AP), CD31 (1:20, Abcam, cat AB28364), and α-SMA (1:200, Sigma, cat C6198), and images were acquired using a Leica SP5 confocal microscope. For quantification of islet composition in explanted grafts, the percentage of glucagon^+^ cells (α cells) and of insulin^+^ cells (β cells) was quantified in each NC or encapsulated islet using ImageJ software (NIH, Bethesda, MD).

### Diffusion Analysis by FRAP

Cell-free capsules were used for diffusion analysis. The supernatant was removed from capsules, placed into a 1.5-ml Eppendorf tube or a 15-ml Falcon tube, and covered with 1 mg/ml solution of FITC-insulin (Sigma), 2-NBD-glucose (Abcam), or FITC-IgG (Sigma). The capsules were incubated at 4°C overnight, and the following day, they were transferred to a clear-bottom dish for fluorescence recovery after photobleaching (FRAP). FRAP was used to characterize molecular diffusion within capsules using a Nikon A1R confocal microscope. FRAP measurements were centralized in the middle of the SC and in the center of each layer for the DC (Supplementary Figure S1). The locations for the measurement were established via a three-dimensional z-stack of the capsule. Once the measurement plane in the *z*-direction was established, ten images were acquired to determine the baseline fluorescent intensities. A circle of 50 µm diameter was selected in the center of the field of view (FOV) as region of interest (ROI). The ROI was bleached for 1 s at full power (65 mW) using a 488-nm laser for FITC-labeled molecules or a 405-nm laser for 2-NBD-labeled molecules. Immediately post-bleaching, time-lapse images were captured each second for 3 min to monitor changes in fluorescence intensity stemming from the diffusion of the fluorescently labeled molecules. Since the amount of fluorescent intensity decreases as a result of laser exposure during time-lapse imaging, a reference area, situated at the periphery of the FOV, was designated to compensate for continuous photobleaching (Supplementary Figure S1, Supplementary Figure S2). The FRAP experiments were repeated in two distinct locations in the middle of at least three different capsules for each molecular probe and each type of capsule. To compare the diffusion rate within the capsule to the diffusion rate in the liquid phase, the FRAP measurements were also acquired in the area surrounding the capsule.

IgorPro (“FRAPcalc” code developed by Kota Miura, 10.5281/zenodo.574203) was used to determine the time point at which 50% of the fluorescent intensity of the photobleaching was restored (“half maximum recovery time,” *τ*). The fluorescent intensity, as a function of time, f(t), was fit to the following Soumpasis diffusion fitting model to obtain *τ*:

$$f\left(t\right)=Ae^{-\frac{\tau }{2t}}\left[I_{O}\left(\frac{\tau }{2t}\right)+I_{1}\left(\frac{\tau }{2t}\right)\right],$$ (1)

where *I*_0_ () is the modified Bessel function of the first kind of order 0 and *I*_1_ () is the modified Bessel function of the first kind of first order to find only parameters A and *τ*. Assuming isotropy, *τ* was then used to calculate the diffusion coefficient:

$$D=\frac{w^{2}}{\tau },$$ (2)

where *w* is the diameter of the ROI. All values were averaged to obtain the final diffusion coefficient value for each sample. The diffusion coefficients were normalized for the size of the unlabeled molecules.

The difference between the diffusion coefficient of the alginate capsules with PLL and the ones without PLL was established using the previously reported technique by Tziampazis and Sambanis (1995). For this, 2% LVM SCs with or without PLL and 1.2% MVG with or without PLL were incubated overnight with 1 mg/ml of FITC-dextran or 2-NBD-glucose. The following day, the capsules were dried to remove the supernatant and added to a 24-well plate (*n* = 3 per condition). Then, 1 ml of Hanks buffer supplemented with Ca^2+^ and Mg^2+^ (HBSS++, Gibco) was added to each well. Samples (75 µL) were taken at different time points (1, 3, 5, 8, 10, 15, 20, 25, 30, 45, 60, 90, 120, 150, 180, 240 min, and 24 h) and added to a 96-well plate for fluorescence reading. HBSS++ (75 µL) was added to each well after each time point. The number of capsules and the diameter of the capsules were measured using a manual differential counter. Diffusion coefficients were calculated using the best fit between the concentration profiles of the experimental solution and the equation describing the solute diffusion out of spheres into solute-free liquid of a constant volume (John, 1975; Verheyen et al., 2019). The values obtained were corrected and normalized for the values obtained with the FRAP technique.

### Computational Analysis

Islet diameters and capsule thicknesses obtained through ImageJ analysis as described in the “Quantification of islet and capsule size” section (Table 4) and diffusion coefficients obtained through FRAP analysis or through the Tziampazis and Sambanis method as described in the “Diffusion analysis by FRAP” section (Supplementary Table S2) were used for our COMSOL Multiphysics-based *in silico* model of insulin secretion. The model (summarized in supplementary information Supplementary Figure S4) was based on the local concentration-based insulin model developed by Buchwald (2011). The concentrations of glucose (*c*_*1*_), oxygen (*c*_*2*_), released insulin (*c*_*3*_), and “local” insulin (*c*_*4*_) were used for convective and diffusive mass transport modeling. For each concentration, the generic diffusion equation of incompressible fluid was used:

$$\frac{\partial c}{\partial t}+\nabla \cdot \left(-D\nabla c\right)=R-u\cdot \nabla c.$$ (3)

Here, *c* is the concentration (mol m^−3^), *D* is the diffusion coefficient (m^2^ s^−1^), *u* is the velocity field (m s^−1^), *R* is the reaction rate, and ∇ is the standard (nabla) operator. Glucose and oxygen consumption and insulin release were assumed to follow Hill-type dependence on the local concentrations (generalized Michaelis–Menten kinetics):

$$R=f_{H}\left(c\right)=R_{max}\frac{c^{n}}{c^{n}+C_{Hf}^{n}\;},$$ (4)

with *R*_*max*_, the maximum reaction rate (mol m^−3^ s^−1^); *C*_*Hf*_, the concentration corresponding to half-maximum response (mol m^−3^); and *n*, the Hill slope characterizing the shape of the response, are the three parameters of the function. The Hill function parameters used in this model were based on published data (Buchwald et al., 2018). First-phase insulin release, $R_{ins,ph1}$ ; second-phase insulin release, $R_{ins,ph2}$ ; and the effect of oxygen availability, $\varphi_{i,o}\left(c_{oxy}\right)$ , were assumed to account for the total insulin release:

$$R_{ins}=\left(R_{ins,ph1}+R_{ins,ph2}\right)\cdot \varphi_{i,o}\left(c_{oxy}\right).$$ (5)

Convection and diffusion models are coupled with the incompressible Navier–Stokes model for Newtonian flow as a fluid dynamics model to describe the velocity field, *u*, that results from convection:

$$\rho \frac{\partial u}{\partial t}-\;\eta \nabla^{2}u+\;\rho \left(u\cdot \;\nabla \right)u+\;\nabla P=F.$$ (6)

Here, ρ represents the density (kg m^−3^), η is the viscosity (kg m^−1^ s^−1^ = Pa s), P is the pressure (Pa, N m^−2^, kg m^−1^ s^−2^), and *F* is the volume force (N m^−3^, kg m^−2^ s^−2^). Aqueous media at 37°C was the flowing media. Incoming media had atmospheric oxygen concentrations (0.200 mol m^−3^) and low or high glucose concentrations (2.2 and 16.6 mM, respectively).

The model was solved as a time-dependent problem, allowing intermediate time-steps, in COMSOL Multiphysics. Mesh (extra-fine) and boundaries used were similar to the previously reported models (Buchwald et al., 2018). Islet and capsule sizes were chosen based on quantifications with ImageJ (average); they were specific for each condition and were compared to standard 150 µm islet size (Table 3).

### Statistical Analysis

Statistical analysis was performed in GraphPad Prism 7.0, using unpaired *t*-test and one-way or two-way ANOVA with post hoc Bonferroni or Dunnet test as adequate. Data are shown as mean ± standard deviation or standard error. Number of animals and independent repeats are indicated for each case; *p* < 0.05 was considered significant. Whenever possible, the investigator carrying out the acquisition and analysis was blinded to the treatment groups.

## Results

We characterized the physicochemical properties and compared the functionalities of the following previously reported islet encapsulation platforms in parallel using NC HIs as a reference:

- PEG CCs
- Alginate SCs: LVM SCs and MVG SCs
- Alginate DCs with PLL: DC + PLL LVM and DC + PLL MVG
- Alginate DCs without PLL: DC LVM and DC MVG.

Capsules were prepared as described in the Methods section in the same lab and on the same day to allow parallel evaluation.

### Islet and Capsule Dimensions

**FIGURE 2:**
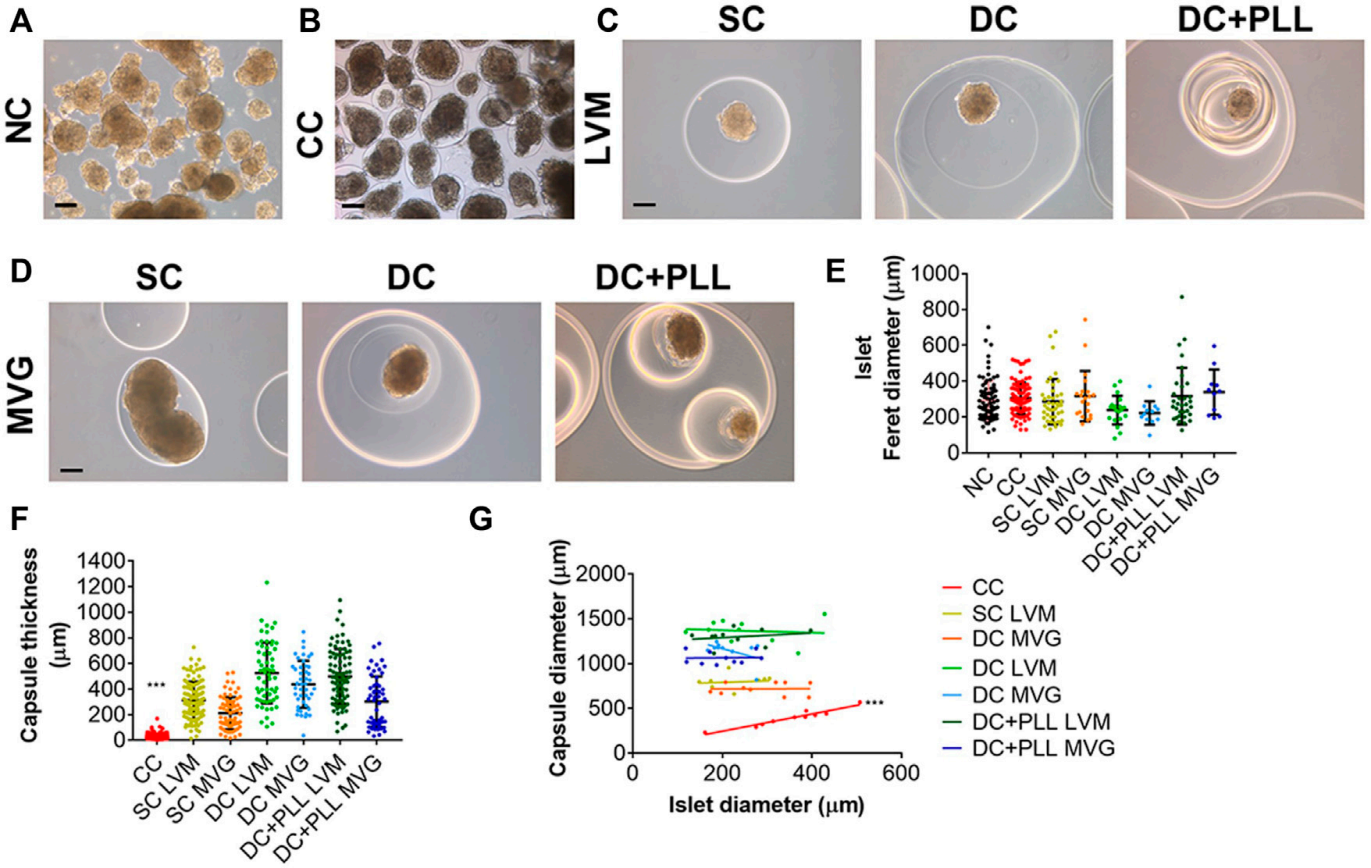
Size characterization of human islets in PEG conformal coatings (CC) or in alginate microcapsules compared to non-coated islets. **(A–D)** Phase contrast images of human islets (HIs) as indicated: **(A)** non-coated (NC), **(B)** encapsulated in conformal coating (CC), **(C)** single (SC) and double LVM alginate capsules with or without PLL (DC + PLL, DC), and **(D)** single (SC) and double MVG alginate capsules with or without PLL (DC + PLL, DC). Scale bars, 100 µm. **(E–F)** Quantification of islet diameter (NC = 7 HI batches, alginate and CC ≥ 3 HI batches) **(E)**, and capsule thickness measured as distance between islet capsule external layer **(F)** (≥3 HI batches per condition), **(G)** Correlation of capsule size with HI size.

We measured the islet size, as Feret diameter, by quantification of phase contrast images (Figures 2A–D), and we compared the size of NC HIs (Figure 2A) (control group) to those of encapsulated (CC, SC, DC) islets (Figures 2B–D), using n ≥ 3 batches of islets per condition. The average Feret diameter of HIs used in this study was 289.32 µm, and there was no significant difference among the groups 1 day after encapsulation (Figure 2E, *p* > 0.2). We also evaluated the thickness of the different capsule formulations and found that all six different alginate capsule groups were significantly larger than the CCs (Figure 2F; Table 4). While SCs were about two times larger than CCs, DCs were about three times larger than CCs. Only three capsule formulations showed comparable thickness: SC LVM and DC + PLL MVG, DC LVM and DC + PLL LVM, and DC + PLL LVM and DC MVG. We found that the diameter of the CC HIs positively correlates with the size of the enclosed islets (*R* ^2^ = 0.9306, *p* < 0.001), indicating that CCs indeed conform closely to the islet shape and size. For all the other capsules, there was no correlation between the diameter of the capsules and the size of the islets (Figure 2G), indicating that these alginate-based encapsulation platforms are not sensitive to islet size.

### Effect of Microencapsulation Platforms on Dynamic GSIS of Human Islets

**FIGURE 3:**
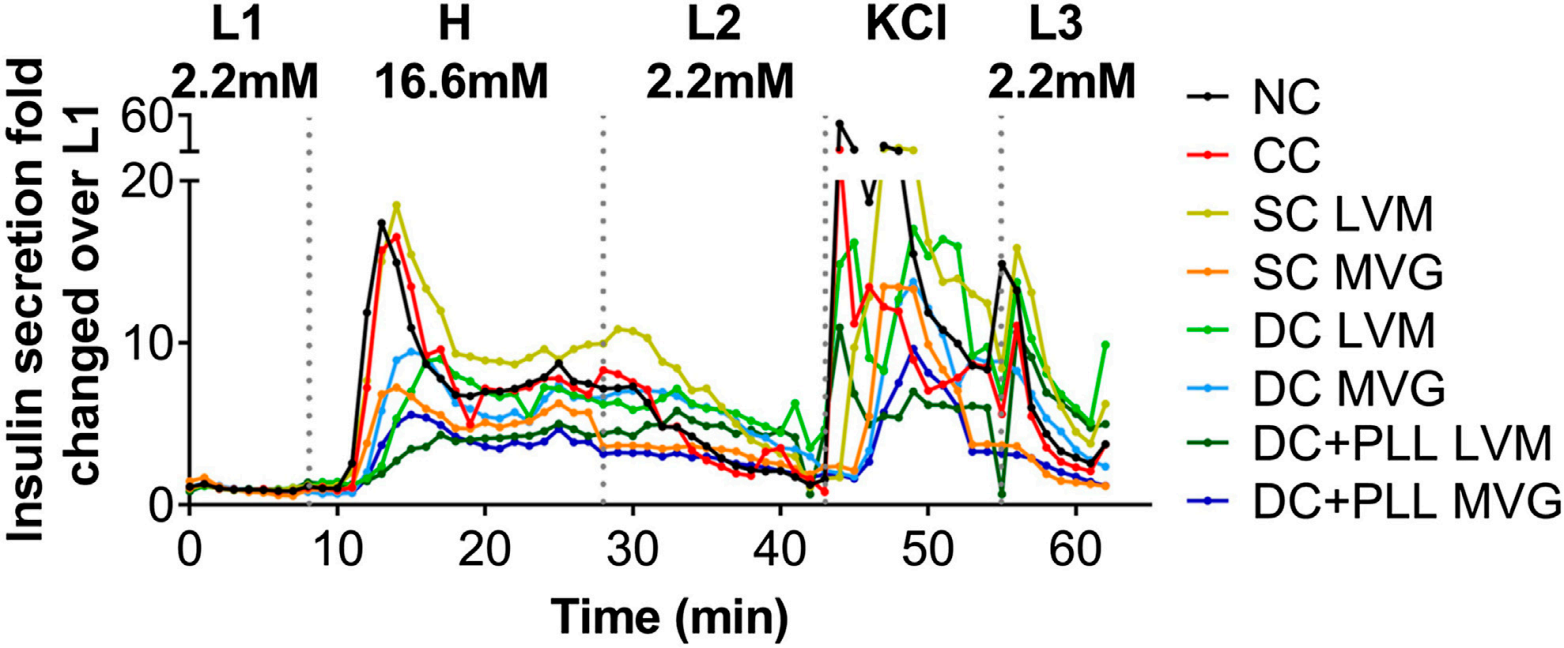
In vitro dynamic GSIS of microencapsulated compared with non-coated human islets. (A) Dynamic GSIS (perifusion assay; 100 μL/min) of human islets (HIs) that are non-coated (NC, black) or encapsulated using conformal coating (CC, red), single alginate MVG capsules (SC MVG, orange), double MVG capsules without (DC MVG, light blue) or with PLL (DC + PLL MVG, dark blue), single alginate LVM capsules (SC LVM, yellow), double LVM capsules without (DC LVM, light green) or with PLL (DC + PLL LVM, dark green). Islets (handpicked 100 IEQs) were perfused for a total of 63 min with 2.2 mM low (L1) glucose (0–7 min), 16.6 mM high (H) glucose (8–27 min), low glucose (L2) (28–42 min), KCl (43–52 min), and low glucose (L3) (53–62 min) as indicated.

To test the effects of capsule formulation on HI functionality *in vitro*, we assessed the insulin release in response to glucose stimulation using high-resolution (1 min) dynamic perifusion assays of microencapsulated HIs (PEG CCs, SC LVM, SC MVG, DC LVM, DC MVG, DC + PLL LVM, and DC + PLL MVG) versus NC controls. We evaluated in parallel the overall time-profile of insulin secretion (Figure 3) and the AUCs during the dynamics of the first phase and second phase (Supplementary Figure S3). The delays in insulin secretion of encapsulated islets were determined by comparing to NC HIs at the peaks of insulin secretion after the L1→H glucose challenge (first phase) and KCl stimulation and the shutdown of insulin secretion after the H→L2 and KCl→L3 transitions (Figure 3). Compared with the NC control, HIs in CCs secreted similar amounts of insulin with small (<1 min) delays in insulin responses. For all six different alginate-based capsule formulations, the thicker the capsule, the more pronounced was the delay (1–5 min) and the reduction in peak height. Importantly, delays in the first phase augmented significantly for DC LVM and DC + PLL LVM/MVG. These delays were caused by the addition of a second layer of alginate and by the presence of PLL, which is known to provide permselectivity and increase capsule biocompatibility (Safley et al., 2013). DCs with PLL showed the most reduced first-phase insulin secretion peaks among all tested capsule formulations. All these indicate that capsule thickness and the presence of PLL are responsible for the observed delays and dampening of glucose-stimulated insulin release of microencapsulated HIs *in vitro*.

### Effects of Microencapsulation Platforms on *In Vivo* Functionality of Human Islets

**FIGURE 4:**
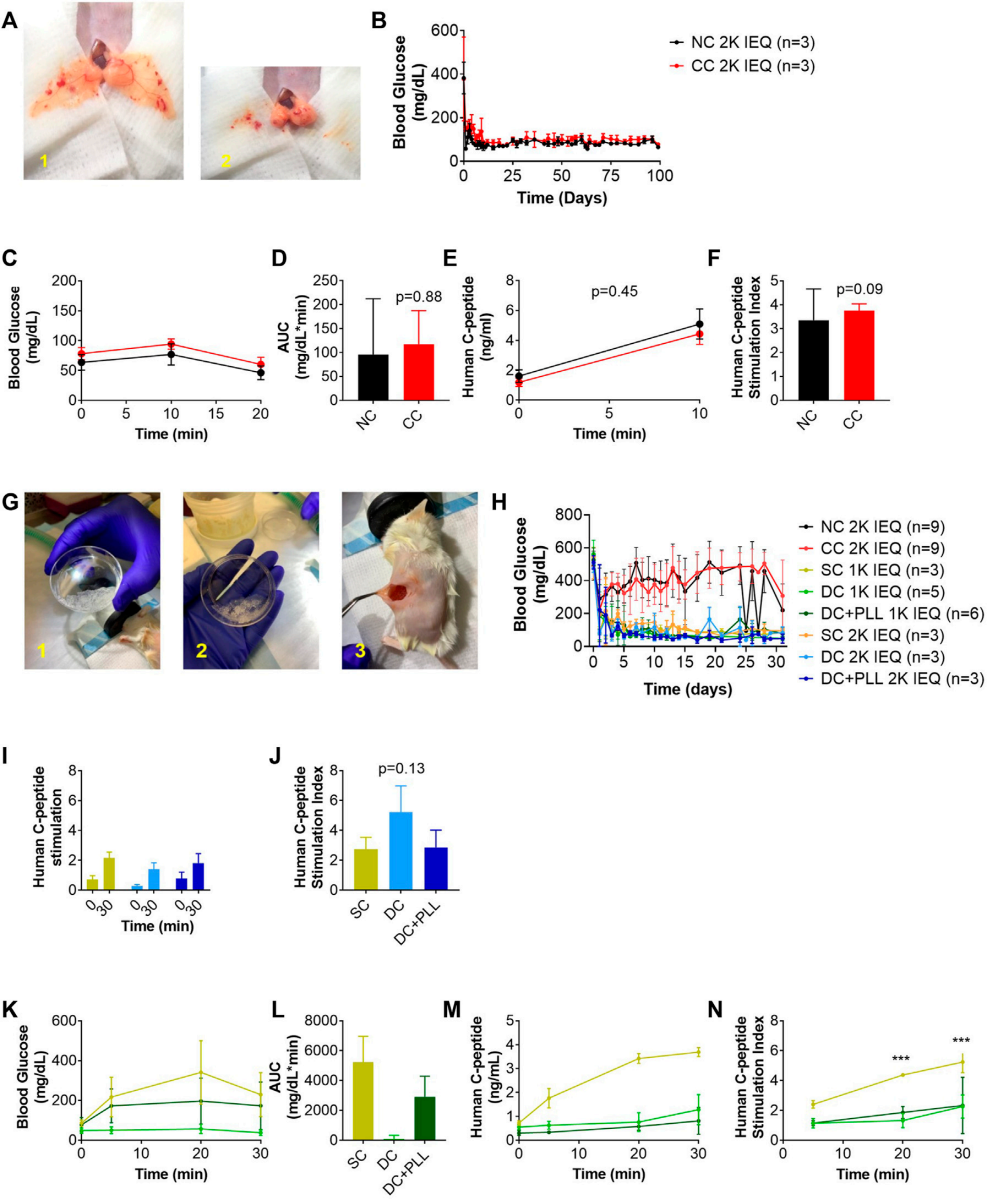
In vivo function of CC and alginate microencapsulated human islets compared to non-coated islets transplanted in their traditional site in diabetic mice **(A–N)**. 2k IEQ human islets (NC HIs, black) and 2k IEQ HIs encapsulated through conformal coating (CC, red) transplanted in the fat pad **(A)** of diabetic NOD-scid mice. 2k IEQ or 1k IEQ HIs encapsulated in single capsules (SCs, yellow) and 2 or 1k IEQ double capsules (DCs) without PLL (light blue and light green, respectively), and 2 or 1k IEQ double capsules (DCs) with PLL (dark blue and dark green, and orange, respectively) transplanted in the peritoneal cavity of diabetic NOD-scid mice **(G)** in two separate experiments. Blood glucose (BG) (B) and glucose tolerance (IPGTT) (C) POD 30 (NC HIs, black; CC, red); AUC **(D)** human c-peptide **(E)**, c-peptide index (NC HIs, black; CC, red) of mice receiving NC or CC **(F)**. BG **(H)**, human c-peptide **(I)**, c-peptide index **(J)**, and IPGTT **(K)** at POD 31; AUC **(L)**; human c-peptide **(M)**, c-peptide index **(N)** of mice receiving NC, CCs, SCs, DCs or DC + PLL. Data are average ±SD for n = 3–6 mice per group.

Following *in vitro* experiments, we performed *in vivo* studies to test whether the observed differences caused by the microencapsulation platforms on *in vitro* GSIS of HIs affect the viability and function of HIs transplanted in chemically diabetic, immunocompromised NOD-scid mice using previously established protocols (Villa et al., 2017; Manzoli et al., 2018; Safley et al., 2020). First, we compared the *in vivo* functionality of NC HIs to CC HIs transplanted in the fat pad (FP) of diabetic NOD-scid mice since both can be accommodated in this confined site (Figure 4A). Median diabetes reversal (MDR) time (Figure 4B) and glucose clearance during GTT (Figures 4C,D) were comparable 30 days after transplantation of 2k IEQ/mouse NC (*n* = 3) or CC (*n* = 3) HIs. Importantly, during the GTT, the human c-peptide of CC HIs increased to levels comparable to those of NC HIs as early as 10 min after glucose stimulation (Figures 4E,F), indicating that CC HIs show no delay *in vivo* in GSIS, confirming observation from the *in vitro* testing (Figure 3 and Table 1).

**TABLE 1:** Times of highest insulin peaks post high glucose (L1→H) and KCl (L→KCl) stimulations (minutes), times of 50% shutdown for insulin secretion during H→L2, and highest peak of insulin secretion (fold change over L1). Shutdown times shown as >43 could not be determined as 50% shutdown was not achieved before the start of the KCl stimulation. For all conditions, n ≥ 3 batches of HIs were analyzed and compared to NC. Asterisks indicate highly significant differences vs. corresponding NC (*p* < 0.001; one-way ANOVA with post-hoc Dunnett tests).

Condition	NC	CC	SC LVM	SC MVG	DC LVM	DC MVG	DC + PLL LVM	DC + PLL MVG
Time (min) of higher peak post high glucose stimulation	13 ± 0.38	14 ± 0.58	14 ± 0.58	13 ± 0.58	16 ± 0.58***	15 ± 0.58***	17 ± 0***	16 ± 0.58***
Time (min) of 50% insulin shutdown post low glucose switch	34 ± 2.27	35 ± 1.15	37 ± 3.06	40 ± 2.51**	>43***	42 ± 1.15***	>43***	42 ± 1.1***
Time (min) of higher insulin peak post KCl stimulation	46 ± 0.82	46 ± 1.52	48 ± 1	48 ± 0.58	51 ± 2***	51 ± 3.79**	49 ± 0.58*	49
Highest peak of insulin secretion (fold change over L1)	17.79 ± 12.39	16.53 ± 13.53	19.04 ± 7.29	6.89 ± 3.49	6.40 ± 4.15	5.03 ± 2.89	9.27 ± 4.29	9.27 ± 3.206

Next, we compared the *in vivo* functionality of capsules with different alginate formulations (SCs vs. DCs with PLL and without PLL) in the intraperitoneal cavity (IP); direct comparison with NC islets was not feasible since we previously demonstrated that NC islets are unable to reverse diabetes after transplantation in the IP site (Villa et al., 2017). We performed two independent studies with two different batches of HIs. In the first study, we evaluated the *in vivo* functionality of HIs encapsulated in SC, DC, or CC to NC HIs. While 2k IEQ of NC (*n* = 3) or CC (*n* = 3) HIs were transplanted in the FP of diabetic NOD-scid mice, 2k IEQ of SC (*n* = 3) or DC (*n* = 3) HIs were transplanted in the IP site. The FP could not be used due to the large volume of these preparations (Figure 4G), as previously reported, and the IP site was used as it is the preferred site for testing these microcapsules, which is relevant for clinical transplantation. In the second study, we transplanted 2k IEQ of NC (*n* = 6) or CC (*n* = 6) HIs in the FP site, 2k IEQ of SC (*n* = 3), 1k IEQ of SC (*n* = 3), or 1k IEQ of DC (without PLL, *n* = 5) or DC with PLL (DC + PLL, *n* = 6) HIs in the IP site of diabetic NOD-scid mice. The different doses of DC in this set of experiments were determined by the large capsule volume that prevented its accumulation even in the IP site. Due to scarce availability of HIs, these studies could not be performed in parallel and were therefore conducted in two different experimental settings, each with its own NC control, though this was performed in the FP site. Blood glucose levels of recipient mice were monitored up to 40 days (first set) and 31 days (second set). HIs in SCs and DCs reversed diabetes 1–3 days after transplantation in the IP site (MDR: 3 days), while NC and CC HIs from this batch did not reverse diabetes by 40 days after transplantation in the FP site, likely due to a suboptimal islet dose for this particular batch of HIs that might be required to reverse diabetes in this site. For SCs and DCs, diabetes reversal was observed at both doses analyzed. However, blood glucose values for alginate capsule recipients were lower than normal human values [70–110 mg/dl (American Diabetes Association, 2019)]: SC 2k 114 ± 43; SC 1k 91 ± 25; DC 2k 83 ± 52; DC 1k 71 ± 35; DC + PLL 2k 63 ± 25; and DC + PLL 1k 93 ± 55 (all mg/dL) with several hypoglycemic episodes (minimum blood glucose): SC 2k 60; SC 1k 56; DC 2k 20; DC 1k 36; DC + PLL 2k 24; and DC + PLL 1k 36 (all mg/dL), and they were more frequent and more severe for DC recipients (Table 2). Human c-peptide levels (Figure 4I) and stimulation indexes (Figure 4J) at 30 min after glucose injection in the first set of experiments were comparable among the different alginate capsules. However, in the second set of experiments, where we also assessed the kinetic of c-peptide release by measuring levels at 5, 20, and 30 min after glucose stimulation, the blood glucose values (Figure 4K), the AUCs (Figure 4L), and the stimulated c-peptide values (Figures 4M, N) were higher in the SC groups than in the DC and DC + PLL groups. These results indicate that, just as we observed in the *in vitro* GSIS experiments (Figure 3 and Table 1), the larger the capsule size, the more delayed and less glucose responsive are the insulin secretions *in vivo* (here, assessed via c-peptide)*.*

**TABLE 2:** Blood glucose (BG) values at d28 and d3–28 of NOD-scid recipients of ALG microencapsulated human islets transplanted in the IP site.

	BG	SC 2k	SC 1k	DC 2k	DC 1k	DC + PLL 2k	DC + PLL 1k
d28	Avg	96	72	43	53	44	105
—	SD	41	11	25	15	12	42
—	low	65	62	20	38	36	66
—	high	142	84	70	72	58	184
d3-28	Avg	114	91	83	71	63	93
—	SD	43	25	52	35	25	65
—	low	60	56	20	36	24	36
—	high	226	203	261	323	149	416

**FIGURE 5:**
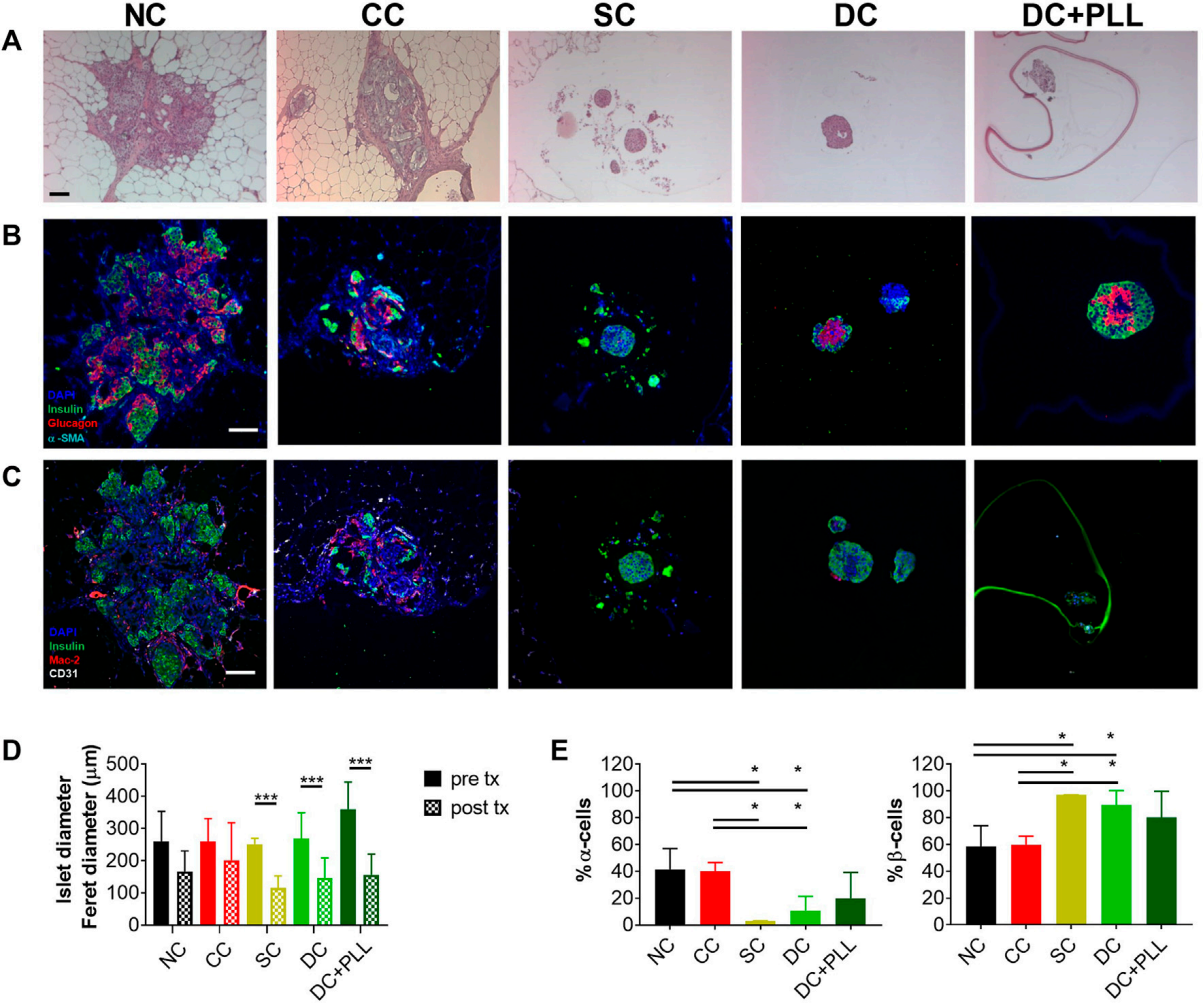
Histological analysis of HI grafts in diabetic NOD-scid mice. Non-coated human islets (NC HIs) and HIs encapsulated through conformal coating (CC), single capsules (SCs), and double capsules with and without PLL (DC + PLL, DCs) were retrieved from the fat pad (NC, CC) or the intraperitoneal cavity (SCs, DCs) of NOD-scid mice 31 days after transplantation. **(A–C)** Representative images of 10x H&E staining **(A)** and ×20 immunofluorescence staining with antibodies against human insulin (green), glucagon (red), and alpha-smooth muscle actin (cyan) **(B)** or against insulin (green), Mac-2 (red), and CD31 (white) **(C)**. **(D–E)** Quantification of HI size pre- and post-retrieval from the fat pad (NC: black, CC: red) and from the intraperitoneal cavity (SC: yellow, DC-PLL: light green, DC + PLL: dark green) **(D)** and of α- and β-cell fractions **(E)**. Data are average ±SD for n = 20 islets from three different preparations.

We analyzed histologically retrieved HI grafts and found that while HIs in CC and NC FP grafts had sizes comparable to their values before transplantation (Figures 5A–D), those in SCs, DC, and DC + PLL IP were significantly smaller than prior to transplantation. We also found that HIs in SC and DC grafts retrieved from the IP site had higher proportions of beta cells (insulin^+^ cells) and a lower proportion of alpha cells (glucagon^+^ cells) in each islet than HIs in NC and CC grafts (Figure 5E), suggesting that islet composition in alginate microencapsulated grafts was altered after transplantation, which could help explain the observed hyperinsulinemia. Absence of Mac−2^+^ cells, including activated macrophages, was observed inside CC, SC, or DC grafts. However, the presence of Mac−2^+^ cells was found around CC grafts, suggesting host innate responses to CC grafts in the FP site.

### Modeling the Effects of Capsule Formulation on Dynamic GSIS

**FIGURE 6:**
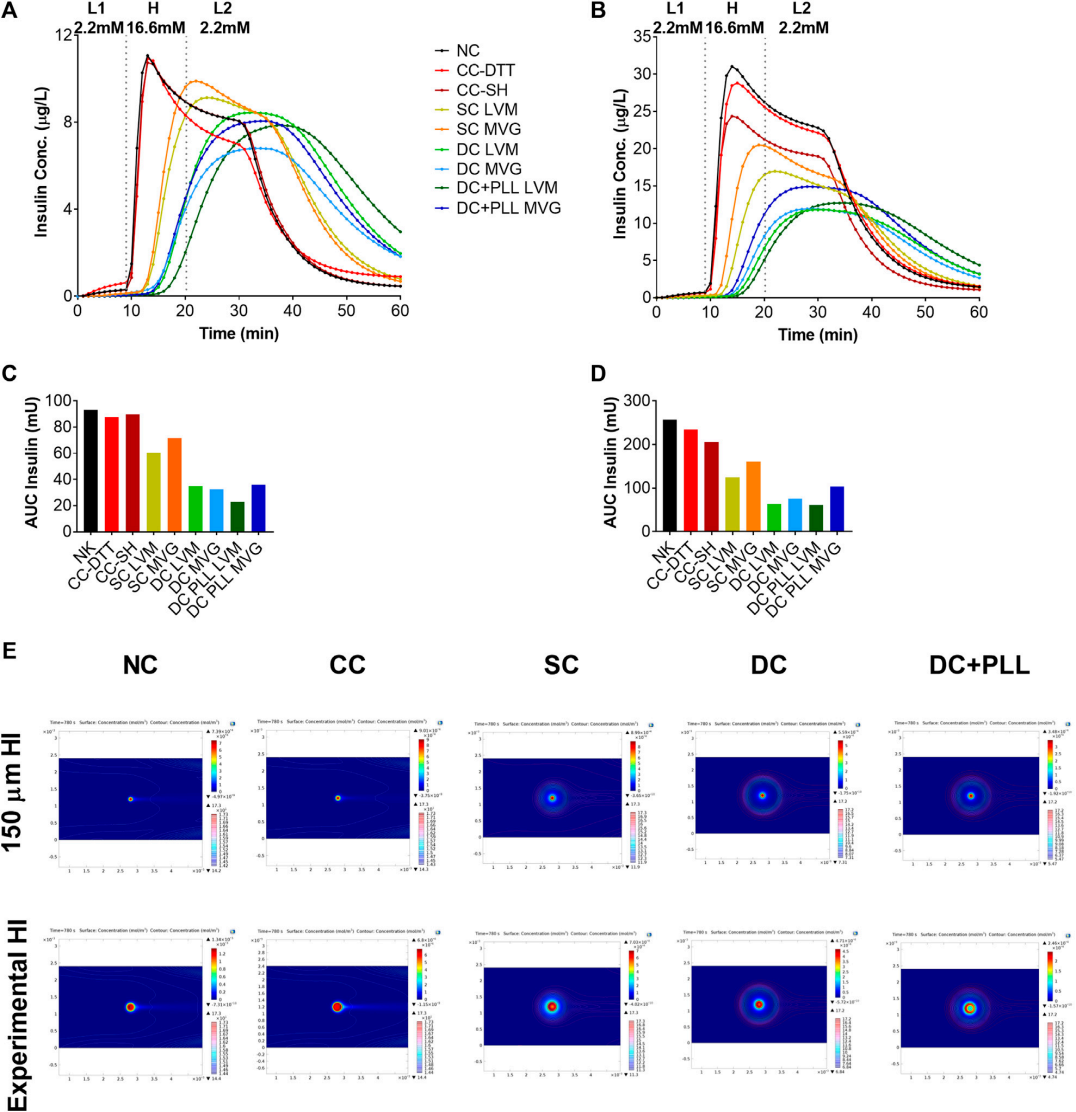
*In silico* glucose-stimulated insulin response of microencapsulated human islets compared with non-coated islets. **(A,B)** *in silico* dynamic GSIS of HIs non-coated (NC, black) or encapsulated in conformal coating (CC, red), single MVG capsules (SC MVG, orange), double MVG capsules with and without PLL (DC MVG PLL, dark blue, and DC MVG, light blue), single LVM capsules (SC LVM, yellow), and double LVM capsules with and without PLL (DC LVM PLL, dark green, and DC LVM, light green). Islets were assumed to be perfused for a total of 60 min with 2.2 mM (mol/m3) low (L1) glucose (0–9 min) followed by 16.6 mM high (H) glucose (10–19 min), and by 2.2 mM low (L2) glucose (20–60 min) as indicated. Simulations were performed using the 150 µm standard islet size **(A)** as well as the experimentally measured average diameters of islets here (Table 1) **(B)**. **(C,D)** Quantification of total units of insulin released as AUC of calculated insulin release profiles shown in panels **(A,B)**. € Concentrations of insulin and glucose of NC and encapsulated HIs at 780 s (time of highest peak of insulin released from NC control) shown as colored surface and contour plots, respectively **(E)**.

COMSOL Multiphysics simulations of dynamic glucose-stimulated insulin secretion of NC and microencapsulated HIs were performed using a previously validated computational model (Buchwald et al., 2018) and the diffusion coefficients measured experimentally here by FRAP analysis (Table 3). As islet size for these simulations, we used both the standard islet equivalent size of 150 µm diameter (Figures 6A,C) and those that were determined experimentally here by an analysis of phase contrast images (Table 4; Figures 6B,D). Insulin release profiles obtained in these simulations strongly depended on the size of the islets and the thickness of the capsules, confirming our *in vitro* (Figure 3 and Table 1) and *in vivo* (Figure 4) observations. In response to glucose increase, NC HIs showed a typical first-phase insulin secretion peak followed by a second-phase plateau. For CCs, we compared PEG formulations with DTT (CC-DTT) or PEG-SH (CC-SH) as crosslinker. Compared with the NC HI control, HIs in either CC formulation secreted similar first- and second-phase insulin amounts with ≤1 min delays in insulin response for the highest first-phase peak and 50% shutdown in insulin secretion when glucose was lowered. In addition, the quantification of the insulin AUCs of NC and CC islets indicated secretion of comparable insulin amounts. For alginate capsules, our model showed a larger delay in insulin secretion during stimulation with 16.6 mM H glucose that was exacerbated by the increase in capsule thickness and further increased when PLL was included in LVM DCs (Figure 6E; Table 5).

**TABLE 3:** Diffusion coefficients obtained through FRAP analysis and used for COMSOL Multiphysics simulations.

D (×10^−10^ m^2^/s) normalized for MW	Water	LVM SC	LVM DC		LVM DC + PLL		MVG SC	MVG DC		MVG DC + PLL	
—	—	—	Inner	Outer	Inner	Outer	—	Inner	Outer	Inner	Outer
INS, *D*_*ins*_	2.58	1.78	1.79	1.96	1.02	1.96	1.74	1.74	1.13	8.00	1.13
GLU, *D*_*gluc*_	8.78	7.31	7.31	5.27	4.02	5.27	6.52	6.52	5.59	5.45	5.59
**D (×10^−10^ m^2^/s) normalized for MW**	**PEG-SH**		**PEG-DTT**	
INS, *D*_*ins*_	1.03		0.91	
GLU, *D*_*gluc*_	8.33		8.80	

**TABLE 4:** Standard (150 µm) islet diameter (condition 1) and diameters of islets obtained through ImageJ quantification (condition 2) and capsule diameters obtained through ImageJ used for the present COMSOL Multiphysics simulations. Values shown as mean ± SD.

	NC	CC	SC LVM	DC LVM	DC + PLL LVM	SC MVG	DC MVG	DC + PLL MVG
Islet diameter (µm) Condition 1	150	150	150	150	150	150	150	150
Capsule diameter (µm) Condition 1	—	350 ± 17	930 ± 144	1,352 ± 236	1,274 ± 211	722 ± 124	1,175 ± 181	913 ± 195
Islet diameter (µm), condition 2	292 ± 109	304 ± 88	286 ± 125	238 ± 77	316 ± 156	317 ± 137	222 ± 62	339 ± 121
Capsule diameter (µm), condition 2	—	355 ± 17	915 ± 144	1,290 ± 236	1,290 ± 211	739 ± 124	1,098 ± 181	944 ± 195

**TABLE 5:** Model calculated time delays in first phase insulin response after L1→H glucose switch for 150 µm standard islet size (condition 1) and for the experimentally measured average HI diameters (condition 2).

Highest insulin peak time (min) post high stimulation	NK	CC	SC LVM	SC MVG	DC LVM	DC MVG	DC + PLL LVM	DC + PLL MVG
Condition 1	13	13	24	24	36	35	39	34
Condition 2	13	13	24	24	36	35	39	34

Thus, these *in silico* simulations (Figure 6) confirmed our *in vitro* results (Figure 3), further validating this COMSOL Multiphysics model. From the AUC analysis, we found that for alginate SCs and DCs, the insulin released by encapsulated HIs continued to increase even after incoming glucose levels were decreased, suggesting that the shutdown of insulin release was also delayed. To further investigate this phenomenon, we analyzed the local concentrations of insulin and glucose in the different capsule formulations at different time points. While glucose levels were rapidly cleared around NC and CC HIs, they remained elevated for longer times around HI encapsulated in all alginate capsule formulations analyzed. Thus, it is likely that the lack of insulin shutdown observed for HIs in alginate capsules is due to diffusional delay of the incoming glucose in the larger capsules leading to prolonged glucose stimulation of alginate encapsulated HIs, which is further exacerbated by the diffusional delays of the released and outbound insulin. This could help explain the *in vivo* results of hyperinsulinemia observed in recipients of alginate microencapsulated HIs.

### Selective Permeability Properties of SC Alginate, DC Alginate, and CCs to FITC-IgG

**FIGURE 7:**
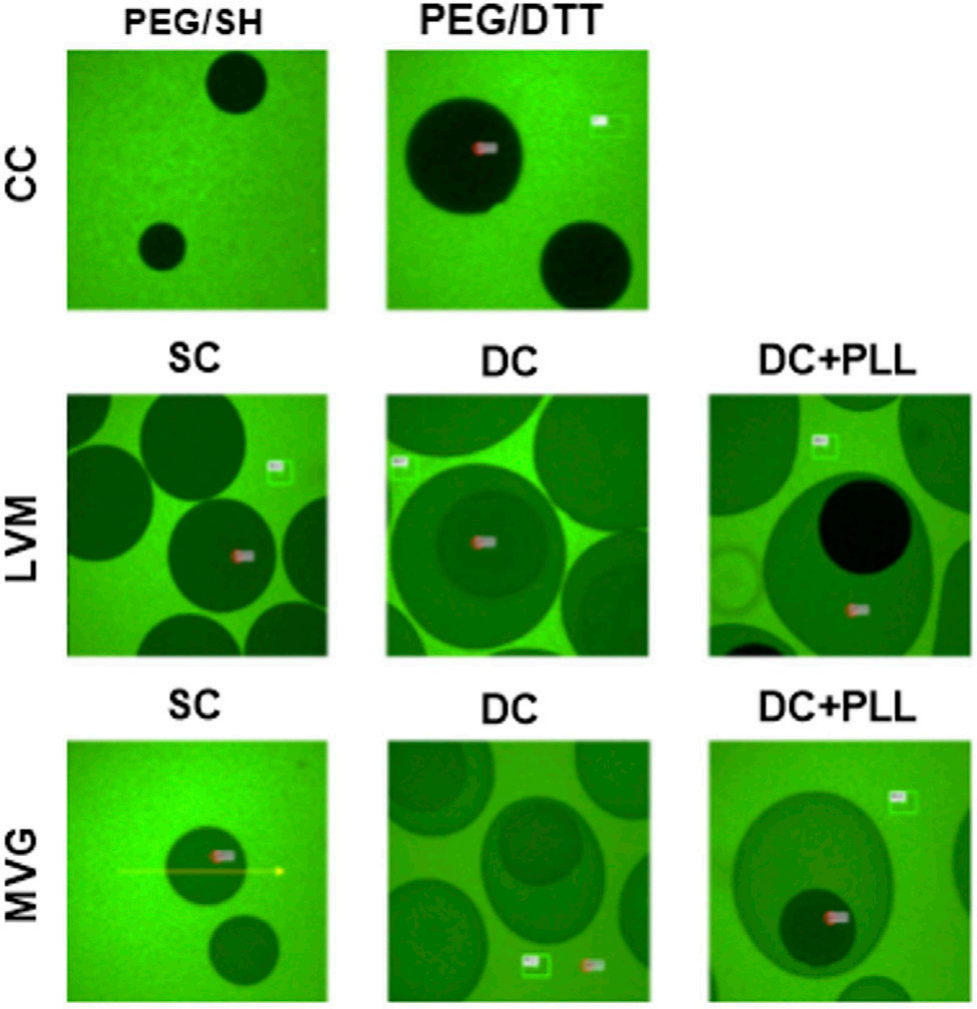
Fluorescence recovery after photobleaching of FITC-IgG. Cell-free CC and alginate SCs and DCs incubated at 4°C overnight with 1 mg/ml FITC-IgG and imaged for FRAP analysis.

We analyzed FITC-IgG diffusion by fluorescence imaging to confirm that while capsules allow free diffusion of glucose and insulin, necessary for islet functionality and quantified by FRAP, they also block IgG diffusion and direct contact with the islets, which is necessary for immunoisolation. We observed that both formulations of cell-free CCs prevented FITC-IgG diffusion. However, in the absence of PLL, FITC-IgG was able to permeate within cell-free alginate capsules (Figure 7).

## Discussion

The main goal of this study was to perform parallel evaluation with human islets of encapsulation platforms that have already been tested in small and large animal models of T1D to identify their strengths and limitations, to inform on which strategies could be integrated to obtain a more functional encapsulation platform for islet transplantation without need of chronic and systemic immunosuppression in T1D (Safley et al., 2013; Tomei et al., 2014; Manzoli et al., 2018; Safley et al., 2018; Safley et al., 2020).

Single alginate microencapsulation has been widely studied. It has been tested as an encapsulation strategy for islet allografts and xenografts in preclinical models and in a few pilot clinical studies, and it has been demonstrated to provide protection to grafted allogeneic islets from the host immune response, but without achieving insulin independence (Omami et al., 2017; Jacobs-Tulleneers-Thevissen et al., 2013). One of the previously reported limitations of single alginate microencapsulation is their lack of selective permeability (permselectivity): IgG can diffuse through SCs made of ultrapure low-viscosity high mannuronic acid (LVM) alginate and ultrapure medium viscosity high guluronate (MVG) alginate (Figure 7). This can lead to cytotoxic damage to the encapsulated islets and poor biocompatibility (Safley et al., 2018; Safley et al., 2020). The addition of a layer of PLL prevents IgG diffusion by enhancing the permselectivity of the capsule. However, since PLL is polycationic, it causes antigen-presenting cell recruitment, attachment on the surface of the capsules, and enhanced immune response (Gu et al., 2019). To prevent this, Weber and coworkers have developed a double LVM alginate capsule, in which the inner capsule is coated with PLL and then re-encapsulated in an outer layer of alginate (Safley et al., 2018). As they observed and we confirmed here for both types of alginate (MVG and LVM alginate), IgG indeed does not diffuse into the inner capsule in the presence of PLL. We found that the islet size, which ranged between 50 and 450 µm prior to encapsulation, did not change significantly 1 day post encapsulation, demonstrating the cytocompatibility of the encapsulation process for the islets. The electrostatic droplet encapsulation procedure produces capsules of fixed size independent of the diameter of the islets (Figure 2G). The size of the capsule depends on the inner size of the nozzle (chosen based on the size of either the islet or the inner SC), on the voltage used (the diameter decreases with increasing voltage), and on the flow rate for alginate solution extrusion (diameter increases with increasing flow rate) (Poncelet et al., 1999). We also found that the addition of PLL shrinks alginate capsules, especially if MVG is used for capsule formulation (Figure 2F).

We confirmed *in silico*, *in vitro*, and *in vivo* that capsule size is an important and limiting factor to consider for glucose-stimulated insulin secretion, which ultimately affects metabolic control of diabetic recipients. Our dynamic perifusion GSIS assays demonstrated that HIs in SCs exhibited first- and second-phase insulin release with a small delay compared to NC HIs, but in DCs, HIs released lower total amount of insulin with increased delay in secretion, indicating that increasing the thickness of the capsules slows islet responses to glucose stimulation. In addition, our *in vitro* studies indicated that the presence of PLL seems to limit glucose/insulin diffusion through the capsules. Comparing DCs and DC + PLL, we observed a more prominent delay in the insulin response for DC + PLL (Figure 3). However, when comparing the two types of alginate, LVM had a higher delay, due to the fact that the capsules were bigger than those with MVG alginate. Although there was a delay in insulin release in the presence of PLL for LVM DCs, PLL did not further delay insulin release from MVG DCs, due to the smaller size of MVG DCs with PLL compared with MVG DCs without PLL (Table 1). The smaller size of MVG DCs with PLL is likely due to a shrinking effect that PLL had on MVG capsules.

The size of the capsules is a limiting factor not only for the functionality of the islets *in vitro* but also for the selection of transplant sites since it increases the total volume of the graft that needs to be transplanted: as the size of the capsules increases linearly, the final islet graft volume increases cubically (Villa et al., 2017). This precludes transplanting a curative dose of alginate capsules in confined and well-vascularized sites such as the FP, which would be favorable for the engraftment of the newly transplanted islets and the enhancement of the metabolic control they can provide (Villa et al., 2017). Therefore, we could not use the FP site for them, and we transplanted alginate capsules in the IP site, as it is traditionally done in the field (Elliott et al., 2005; Villa et al., 2017; Bochenek et al., 2018; Safley et al., 2018; Safley et al., 2020). Accordingly, one limitation of the study is the impossibility to compare directly NC and CC islets to alginate-based formulations due to the different allowable transplant sites. Another limitation is the need to reduce the dose of DCs transplanted in the IP site due to their larger volume than SCs. However, these necessary protocol modifications are relevant for clinical application since each capsule design was tested in their applicable transplant site and dose. Thus, we decided to compare NC to CC islet performance in the FP site and SCs, DC with PLL, and DC without PLL in the IP site. *In vivo* diabetes reversal with the alginate capsules was immediate; however, DCs caused hypoglycemia. Our findings suggest that blood glucose levels of recipients of alginate microencapsulated HIs in the IP site are maintained at lower levels than normal human ones with several hypoglycemic episodes, indicative of possible hyperinsulinemia, which was not observed in the recipients of NC HIs in the FP site (Park et al., 2012).

Furthermore, although encapsulation does not modify islet size prior to transplantation, islet size decreased significantly post transplantation, and the proportion of glucagon-producing alpha cells diminished, suggesting that because high glucose levels tend to remain elevated around alginate islets for prolonged times, as indicated by our *in silico* results, they might downregulate glucagon cells impairing metabolic control, which is usually obtained with 35–40% of alpha cells in the islets (Rorsman and Braun, 2013).

Our *in silico* analysis in COMSOL Multiphysics using the computational model previously established (Buchwald et al., 2018) with additional parameters obtained experimentally here for each condition (Table 5) confirmed that alginate capsules release insulin with a delay that increases with the thickness of capsules and the presence of PLL. They also indicated that glucose remains longer inside the larger capsules causing overexposure of the islets to local high glucose and stimulating them to release insulin for more prolonged times, and possibly even triggering phenotypical changes in the islet composition over time. This could explain why alginate DCs that did not produce physiological insulin response *in vitro* caused hypoglycemia in the transplanted mice *in vivo* (Figure 4): prolonged exposure to high local glucose concentrations exhausts the insulin-producing β cells and shuts down the glucagon-producing α cells. Furthermore, we observed that retrieved alginate encapsulated islets were significantly smaller in size compared to pre-transplantation. This could be due to the poor oxygenation of the IP site that, together with the larger size of these capsules, can lead to islet necrosis (Manzoli et al., 2018). Although these results may seem discouraging, it is important to underline that this is not an alginate-based effect. Through *in silico* simulations, we saw that by decreasing the alginate capsule size, the response of the islets to glucose stimulation reverts to a normal profile, even if the PLL layer is maintained (Supplementary Figure S5). Importantly, alginate capsules showed no cell attachment into the IP site, confirming the high biocompatibility of such encapsulation platforms for islet transplantation (Safley et al., 2020).

The CC technology was developed specifically to address the size issue associated with traditional microencapsulation technologies. CC allows formation of capsules that conform to the islet shape and size with similar thickness irrespective of islet size (Tomei et al., 2014) (Figure 2G) and graft volumes comparable to those of NC HIs allowing transplantations in the same sites, including confined, well-vascularized sites such as FP. This CC platform has already been shown to be immunoisolating for allogeneic islets transplanted in the FP of C57BL/6 mice without immunosuppression and to support >100 days survival without immunosuppression after transplantation (Manzoli et al., 2018). Diffusivity studies here indicated that IgG does not penetrate the CC layer, confirming its immunoprotective properties (Figure 7). Moreover, the encapsulation process is not harsh on islets: islet size is comparable between CC and NC HIs, and functionality of CC islets is maintained. Following *in vitro* glucose challenge, CC and NC HIs had similar first- and second-phase insulin release profiles, with only a very brief (≤1-min) delay in insulin response for both the high peak and the shutdown and no decrease in peak heights or amount of total insulin secreted (AUC). These results suggested that smaller capsules are needed for more physiological insulin secretion and proper metabolic control. We previously demonstrated that CCs reversed diabetes *in vivo* with different islet sources (Tomei et al., 2014; Manzoli et al., 2018; Stock et al., 2020). Here, we transplanted NC and CC HIs in NOD-scid mice either in the mammary or the epididymal FP. We found that the blood glucose and c-peptide levels with CC are comparable to NC islets (Figures 4A–F), indicating that the results that we observed *in vitro* are confirmed *in vivo*. We observed that NC and CC HIs responded similarly whether the grafts were functional or nonfunctional; we found that there is a HI batch-to-batch variability. For this reason, we believe that it is necessary to perform functionality and viability assays prior to encapsulation and transplantation and to base the islet dose on the quality of each islet batch determining the specific islet dose required for each batch for diabetes reversal.

Islet size in CC grafts remained in the same range as prior to transplantation, indicating that CC islets are well oxygenated. In addition, using the same batch of islets for transplantation, post transplantation the proportion of α cells in NC and CC islets is higher than that in the alginate capsules, while the proportion of β cells is lower. This imbalance compared with NC islets could explain why alginate encapsulation of HIs tended to result in hypoglycemia in NOD-scid mice. Differences in diabetes reversal rate between alginate capsule grafts transplanted in the peritoneal cavity and NC and CC grafts in the FP for the same islet batch could be explained by higher local inflammatory events in well-vascularized sites such as the FP (which could decrease graft functionality) compared with the avascular IP cavity (Vaithilingam et al., 2017). Finally, we observed that Mac−2^+^ cells were present around the CC grafts transplanted in the FP site, indicative of host responses to the encapsulated graft. These innate immune cells could proliferate and, in the process of phagocyting CCs, could form foreign-body giant cells that engulf the capsules, causing chronic inflammation and islet graft loss (Anderson et al., 2008; Sheikh et al., 2015) if left untreated.

## Conclusion

In conclusion, to provide reliable immunoprotection, SCs based on alginate need to be coated with PLL and a second layer of alginate (DC + PLL) for optimal permselectivity, resulting in increased biocompatibility and improved immunoprotection. On the other hand, the large DC size and the PLL coating limit their applicability, not only limiting transplantation to sites that can accommodate large volumes of capsules, like the intraperitoneal cavity, but also dampening and delaying the insulin response. Finally, we believe that the large size and the resulting prolonged exposure to elevated glucose levels can cause the shutdown of glucagon-producing α cells, resulting in hyperinsulinemia, poor glycemic control, and increased frequency of hypoglycemic episodes. Both *in vitro* and *in silico* studies indicated PEG CCs to be ideal for metabolic control and suitable for transplantation in the same sites as NC HIs, including confined and well-vascularized sites, due to their smaller diffusion barriers and smaller sizes. While permeable to glucose and insulin, CCs were found to be efficient in protecting the islets from IgG diffusion. However, we saw high variability *in vivo*, possibly due to suboptimal biocompatibility of these smaller capsules and differences in the quality of HIs among different isolation batches.

We believe that the limitations of the capsules presented here are not related to the choice of the material used (alginate or PEG), and they can be addressed by minimizing the size of alginate DCs to improve their metabolic control capability and the use of CCs in combination with a localized anti-inflammatory drug delivery platform to improve their biocompatibility. A smaller sized DC + PLL may provide better metabolic control and also allow transplantation in confined and well-vascularized sites. As demonstrated *in silico*, DC + PLL with the same size as CCs can provide physiological insulin secretion and metabolic control (Supplementary Figure S5). Smaller DC + PLL also would allow transplantation into the same sites than NC islets including confined sites such as the FP. To maintain the biocompatibility of smaller DC + PLL and improve those of CCs, localized and controlled delivery of anti-inflammatory drugs could be used (Velluto et al., 2021). To improve the *in vivo* variability of CCs, the quality of the islets should be assessed prior to encapsulation, and a correspondingly adjusted dose of IEQ should be used to ensure diabetes reversal in the recipients.

## Data Availability

The raw data supporting the conclusion of this article will be made available by the authors, without undue reservation.
