# Supplementary material for: Parallel Evaluation of Polyethylene Glycol Conformal Coating and Alginate Microencapsulation as Immunoisolation Strategies for Pancreatic Islet Transplantation
Source: Front Bioeng Biotechnol. 2022 May 16;10:886483. doi: 10.3389/fbioe.2022.886483 (PMC9149081; doi:10.3389/fbioe.2022.886483)
Supplement: Supplementary file 1 [file DataSheet1.PDF]

SUPPORTING INFORMATION

Table S1: Diffusion coefficients of water and NC HIs used for COMSOL Multiphysics simulations [19].

Table S2: Diffusion coefficients of FITC-IgG obtained through FRAP analysis.

Table S3: Diameter of islets, and radius of capsules used for COMSOL Multiphysics simulations.

Figure S1: Single and double alginate capsules imaged during FRAP analysis

Figure S2: Schematic of Fluorescence recovery after photobleaching (FRAP) protocol

Figure S3: Area under the curve (AUC) of in vitro dynamic GSIS of microencapsulated compared to non-coated human islets

Figure S4: Schematic of COMSOL Multiphysics model

Figure S5: Comparison of *in vitro* glucose-stimulated insulin secretion of small size DC+PLL to NK HIs.

**Species/media**	**Water (x10^{-9} m^2/s)**	**Tissue (islet) (x10^{-9} m^2/s)**
Oxygen, D_{oxy}	3.0	2.0
Glucose, D_{gluc}	0.9	0.3
Insulin, D_{ins}	0.15	0.05

Table S1: Oxygen, glucose, and insulin diffusion coefficient used for water and islet tissue in the COMSOL Multiphysics simulations, D [m^2·s^{-1}].

**D (x10^{-11} m^2/s) normalized for MW**	**Water**	**LVM SC**	**LVM DC**		**LVM DC+PLL**		**MVG SC**	**MVG DC**		**MVG DC+PLL**	
			**Inner**	**Outer**	**Inner**	**Outer**		**Inner**	**Outer**	**Inner**	**Outer**

FITC-IgG, D_{IgG}	0.75	2.17	2.50	3.13	2.50	3.38	2.81	2.93	3.54	2.93	3.08

**D (x10^{-11} m^2/s) normalized for MW**	**PEG-SH**	**PEG-DTT**
FITC-IgG, D_{IgG}	9.73	4.84

Table S2: Diffusion coefficients of FITC-IgG obtained through FRAP. Cell-free capsules were used for diffusivity studies. Capsules were dried to remove any residual supernatant and placed into a 1.5 mL Eppendorf tube or a 15 mL Falcon tube and covered with 1mg/mL FITC-IgG (Sigma). Capsules were left incubating at 4°C overnight, and the following day, they were transferred to a clear bottom dish for FRAP measurements.

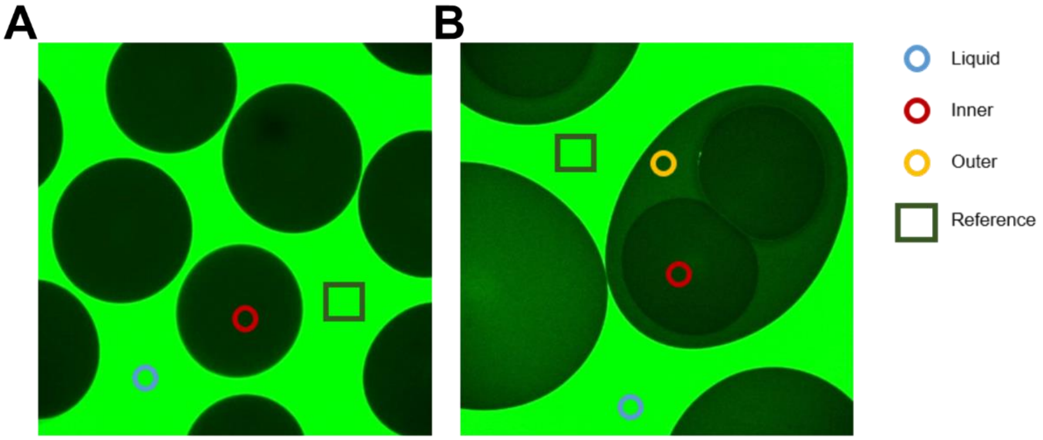

Figure S1. Single and double alginate capsules imaged during FRAP analysis

Confocal images of a single hydrogel (LVM) capsule (SC, **A**) and a double hydrogel (LVM) capsule (DC, **B**) with the designated regions of interest (ROIs): red, yellow, and blue circles of 50µm diameter and green square as reference.

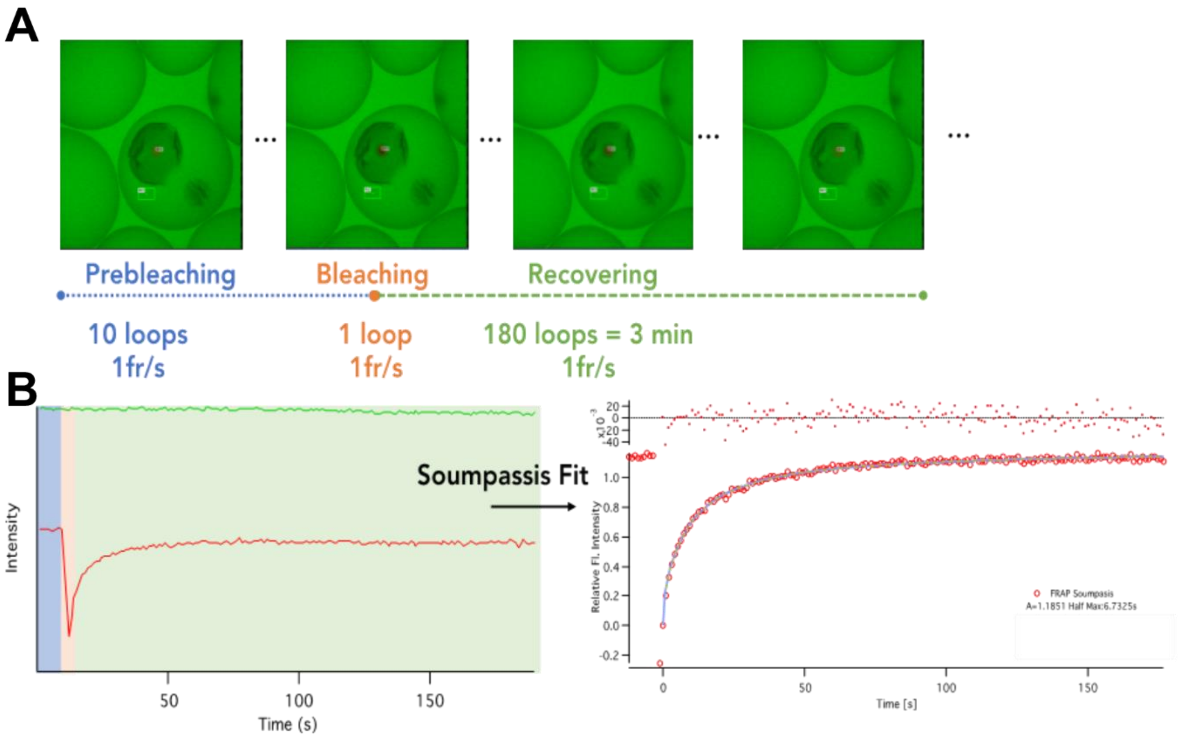

Figure S2. Schematic of Fluorescence recovery after photobleaching (FRAP) protocol. Time-lapse images of the full field of view during FRAP (**A**). Quantitative monitoring of fluorescence intensity within the ROIs and the Soumpassis fit of the fluorescence intensity recovery inside the inner capsule (**B**).

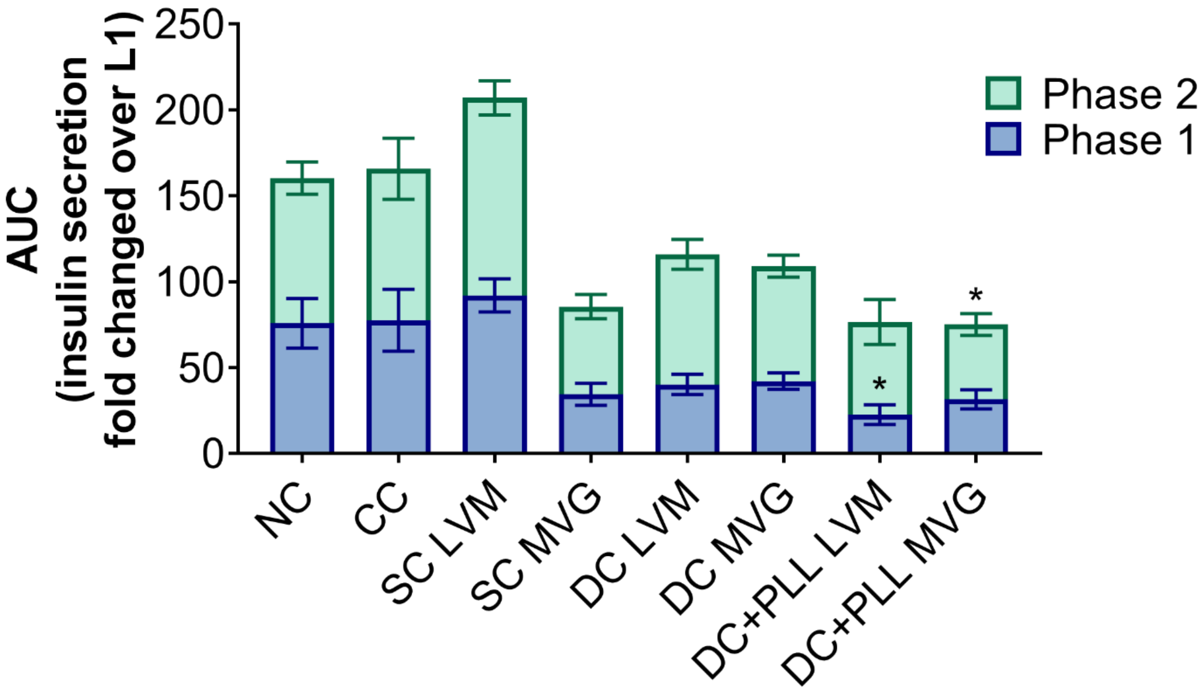

Figure S3. Area under the curve (AUC) of *in vitro* dynamic GSIS of microencapsulated compared to non-coated human islets

Areas under the curve (AUCs) for glucose stimulated insulin secretion profiles of the different conditions and separated into phase 1 (11 to 19 min) and 2 (20 to 32 min). Asterisks denote significant differences versus the NC HIs. For all conditions, n>3 batches of HIs were analyzed.

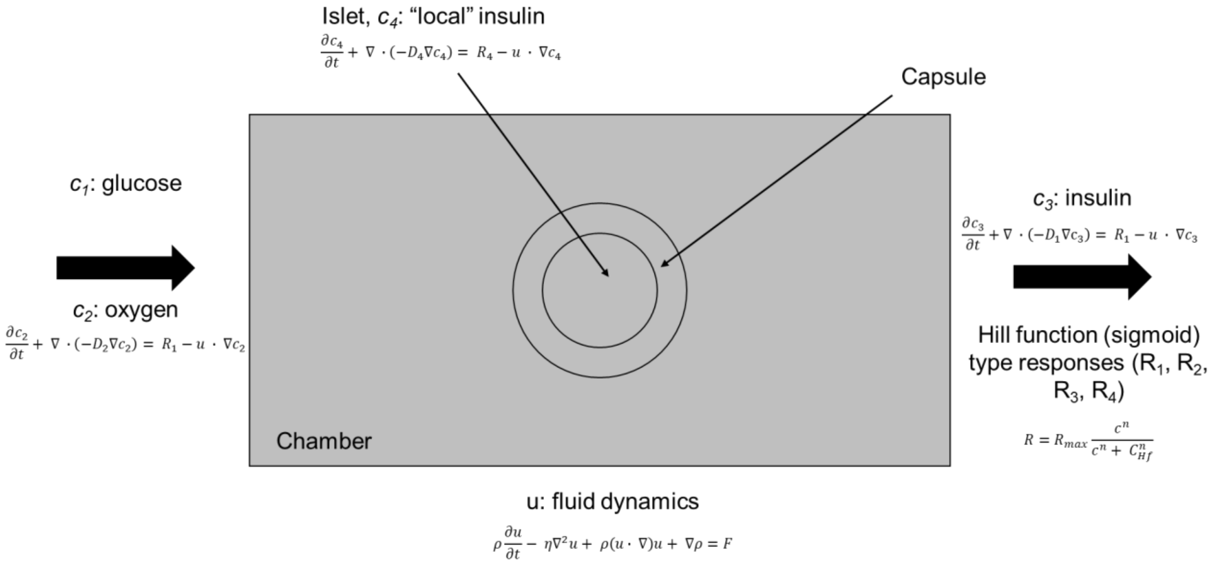

Figure S4. Summary of the COMSOL Multiphysics model used for *in silico* evaluation of glucose-stimulated insulin release.

**Diameter islets (µm)**	**NC**	**CC**	**DC+PLL LVM**	**DC+PLL MVG**
Condition 1	150	150	150	150
Condition 2	292	304	316	339
**Diameter capsules (µm)**	**/**	**CC**	**DC+PLL LVM**	**DC+PLL MVG**
Inner		355	355	355
Outer			409	409

Table S.3: Diameter of islets, and radius of capsules used for COMSOL Multiphysics simulations

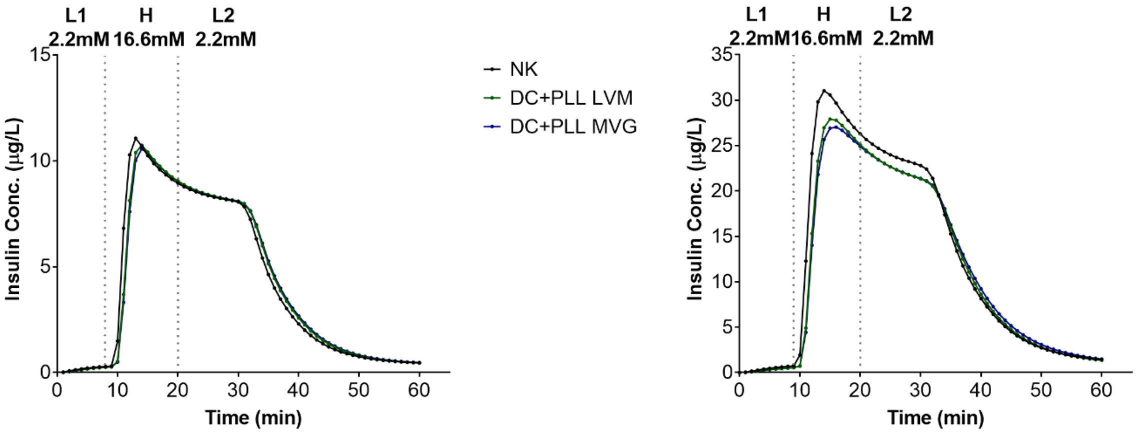

Figure S5: Comparison of *in silico* glucose-stimulated insulin secretion of small size

DC+PLL to naked human islets. COMSOL Multiphysics simulations of dynamic glucose-stimulated insulin secretion of NK (black) and HI encapsulated in small DC+PLL (dark green LVM; dark blue MVG). Islets were assumed to be perfused for a total of 60 minutes with 2.2 mol/m^3 low (L1) glucose (0-540 s) followed by 16.6 mol/m^3 high (H) glucose (600-1140 s), and by 3 mol/m^3 L2 glucose (1200-3600 s) as indicated. Simulations were run using the 150 µm standard islet size (**A**) and using the experimentally measured average diameters of islets (**B**). We demonstrated that in small DC+PLL the response of the islets to glucose stimulation reverts to a normal profile, even if the PLL layer is maintained.
